# The Impact of a National Surgical Mask Wearing Policy on COVID-19 Transmission in Haemodialysis Units in the Republic of Ireland

**DOI:** 10.1016/j.ekir.2020.12.031

**Published:** 2021-01-01

**Authors:** Donal J. Sexton, Patrick O’Connor, Eileen McBrearty, George Mellotte

**Affiliations:** 1 Trinity Health Kidney Center, St James’s Hospital, School of Medicine, Trinity College Dublin, Dublin, Ireland; 2 The National Renal Office, Health Services Executive of Ireland

In late 2019 a novel betacoronavirus named SARS-CoV-2 was reported in China, and subsequently spread rapidly to cause a global pandemic.^1^ SARS-CoV-2 is primarily spread through respiratory droplets typically released by coughing, sneezing, breathing or speaking and may be aerosolized by singing, ventilation or the use of nebulisers.^1^ The most common symptoms of COVID-19 infection include fever, cough, fatigue, anorexia, myalgia and diarrhoea,^2^ however severe illness occurs in a subset of individuals and usually begins approximately 1 week after the onset of symptoms.^2^ Patients with End Stage Kidney Disease (ESKD) have been identified internationally as one such vulnerable subgroup at risk of severe COVID-19 infection and complications.^3^^,^^4^

In Ireland the Health Protection Surveillance Centre (HSPC) confirmed the first case of COVID-19 in the Republic of Ireland on 29 February 2020. The governance of the care of all patients with end stage kidney disease treated with maintenance dialysis in Ireland lies with the Health Services Executive (HSE) of Ireland and specifically the National Renal Office (NRO) in the HSE. The HSPC and the NRO issued a joint guidance document regarding the management of dialysis patients during the COVID-19 pandemic on March 16 2020.^5^ This included strategies on how to identify patients at risk, provide isolation facilities and personal protective equipment, guidance on 2-meter social distancing, patient transport provision and a stipend to facilitate self-driving of dialysis patients to and from the dialysis center. At the time of the first wave of the pandemic in the Republic of Ireland, there were 1925 adult haemodialysis patients distributed across 23 haemodialysis units nationally.

Asymptomatic and pre-symptomatic spread of the virus represents a significant challenge to the containment of SARS-CoV-2 infection, particularly in environments such as dialysis units. Measures such as physical distancing, limiting public gatherings, large-scale stay at home orders, and national lockdowns are now known to curb COVID-19 transmission.^6^ However at the time of the pandemic first wave onset in Ireland, good quality evidence endorsing the efficacy of facemasks were lacking. The NRO recognized that dialysis patients were not only vulnerable to COVID-19 complications but also could not adequately self-isolate by virtue of the fact that they had to present to the dialysis centers multiple times per week. In addition the NRO was cognizant of suggestions arising from the pandemic in Italy that dialysis units may have been associated with transmission from healthcare settings back into the community. Despite the lack of evidence at the time, since it was considered a low cost intervention associated with empiric logic, the NRO instituted a mandatory surgical mask wearing policy for dialysis patients and dialysis healthcare workers for the duration of dialysis sessions on 7 April 2020. With the support of the HSE Medical Devices Critical Assessment Group and HSE procurement, the NRO distributed digital thermometers and surgical masks to all haemodialysis patients in the Republic of Ireland from 7 April 2020 onwards.

Dialysis patients were requested to wear these surgical masks prior to arriving at the dialysis unit and to continue wearing the masks throughout the entire time in the dialysis unit. In addition eating on dialysis was not permitted to limit time without masking, and healthcare workers were instructed to wear surgical masks at all times while in clinical areas.

We questioned whether the introduction of universal surgical mask wearing in all dialysis units nationally was associated with a decline in COVID-19 transmission in dialysis patients in Ireland.

## Results

As of 1 January 2020 there were 4747 adult patients with ESKD in the Republic of Ireland, conisting of 1925 patients on haemodialysis, 256 on home therapies (Peritoneal Dialysis and Hemodialysis) and 2566 kidney transplant recipients. On 30 August 2020, the absolute death rate from COVID-19 in patients with ESKD was 0.7%. (21 of 4747). This can be further subdivided into treatment categories as follows: 1.4% of all haemodialysis patients died due to COVID-19 (27 of 1925), 0.15% of transplant patients (4 of 2566) and none of the home therapy cohort died. By June 2020 the haemodailysis cohort had risen from 1925 to 1996 nationally while also incorporating deaths over this period (deaths due to COVID-19 and independent of COVID-19). Each dialysis unit nationally confirmed their compliance with the surgical mask wearing policy while submitting updated reports on cases from individual units throughout wave 1 of the pandemic on a weekly basis. At the time of writing (30 August 2020) there has not been a case of COVID in a dialysis patient in the Republic of Ireland since 19 May 2020.

### Results of Non-Linear Regression

**Figure 1:**
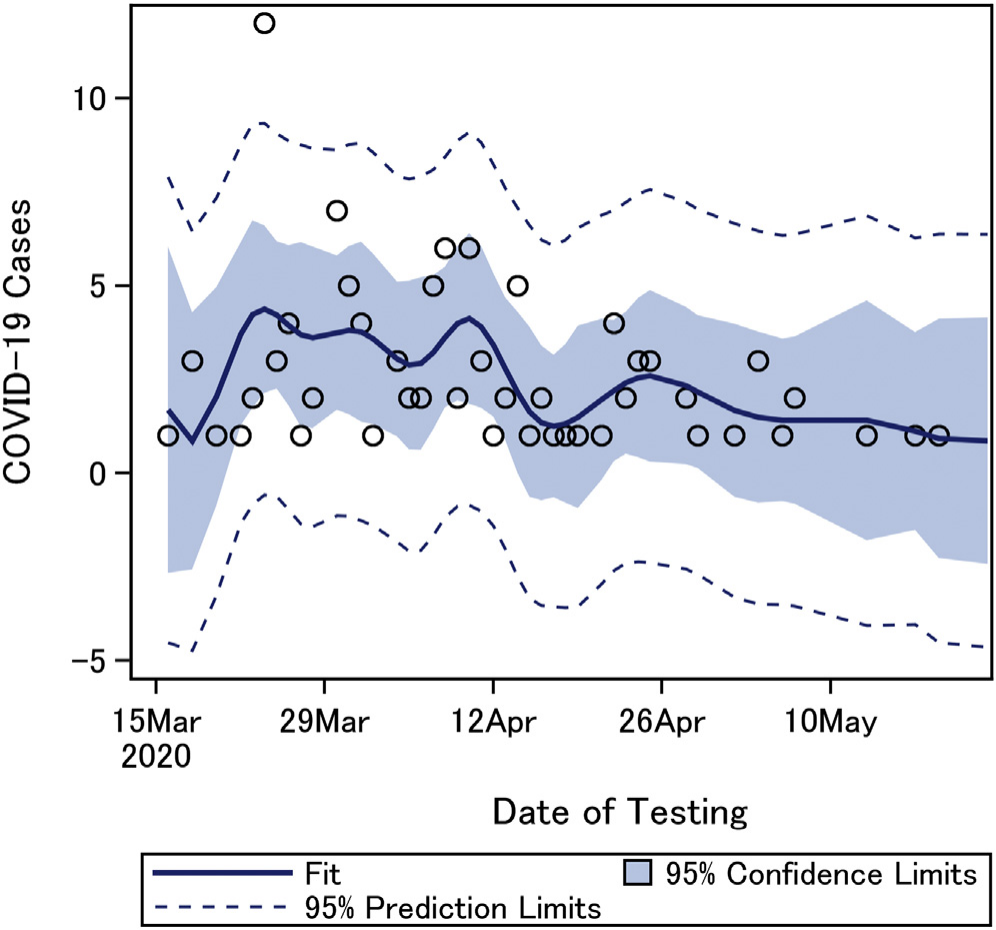
Non-linear regression analysis of positive COVID-19 testing in dialysis patients in Ireland. Note a significant decline following the period of mask introduction.

There was a significant net slope change after the period of mask policy introduction. Median positivity rate in the pre-intervention period was 0.16% (IQR 0.15%), and 0.10% (0.05%) in the post intervention period (after mask introduction) p = 0.02 (Figure 1). Results were not different while accounting for a lag effect of 5 to 7 days following the interventon dates for either intervention.

### Results of Forecasting Procedure

**Figure 2:**
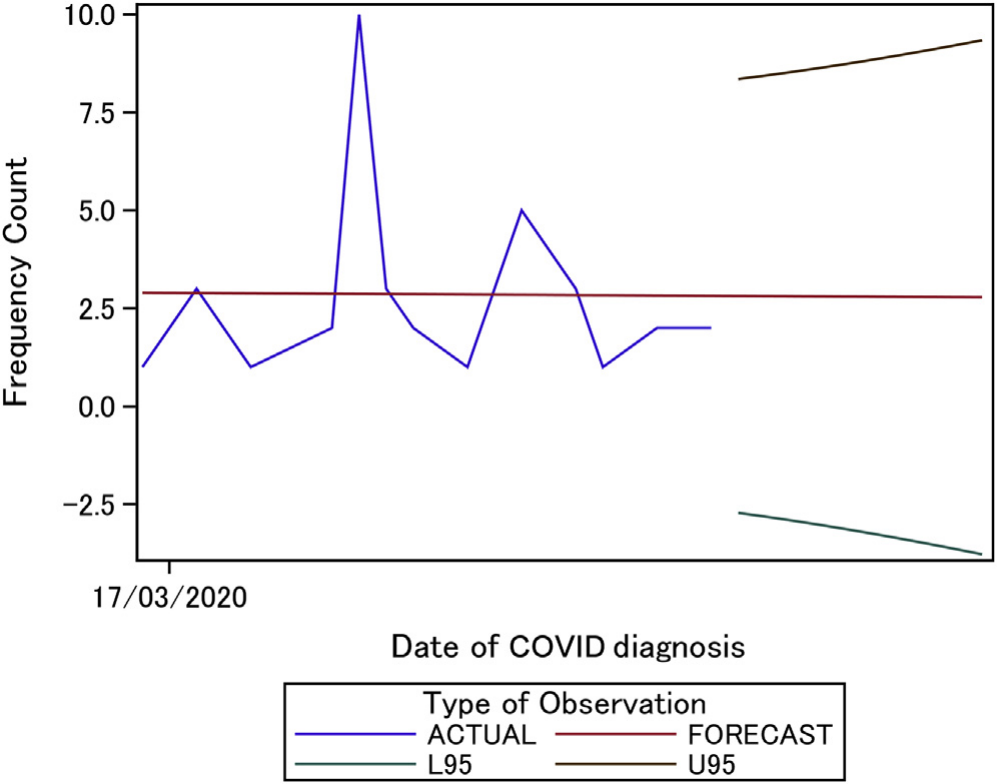
Forecasting based on daily COVID-19 positive cases in haemodialysis patients prior to 7 April 2020, marking the introduction of a national surgical mask policy in haemodialysis units in Ireland. Frequency count represents total number of cases per day from 17 March 2020 onward. Based on case count prior to the surgical mask policy, forecasting suggested a relatively static case count.

Forecasting suggested a projected static COVID-19 positive case rate for the period following 7 April 2020 (Figure 2). It would appear that the introduction of universal mask policy altered this projected course since cases fell in the intervention period defined by national universal masking in dialysis units rather than being static. Results were not different while accounting for a lag effect of 5 to 7 days following he interventon dates for either intervention.

## Discussion

At the time of writing there is now emerging evidence from multiple studies worldwide that wearing facemasks does indeed appear to curb the spread of COVID-19.^7^ However, there are few reports pertaining to the application of surgical mask wearing policies to the specific environment of the haemodialysis unit. In Ireland, the NRO, mandated a national policy of surgical face mask wearing by patients and healthcare staff in all dialysis units nationwide on 7 April 2020. This was due to cognizance that dialysis patients were vulnerable to complications from COVID-19 infection and also evidence from Italy of possible transmission from dialysis units out into the community.^3^^,^^4^ In addition, dialysis patients could not shelter in place due to the fact that they must attend the dialysis unit three times per week often while sharing transport with other dialysis patients.

Our study design precludes the identification of definitive causal relationships. While it appears as though the COVID-19 case rate declined progressively after the introduction of a mandatory surgical mask wearing policy in HD units nationwide, we cannot conclude this definitively and it is possible that other factors may have also contributed. Our reported trends were consistent while also accounting for a two weeks period following the introduction of the COVID-19 strategy measures in the general population on 12 March 2020. Other studies have found similar results from mask wearing policies in healthcare settings other than dialysis units, including an incremental benefit of both healthcare workers and patients wearing surgical masks.^8^ However, data pertaining to dialysis units is lacking.

The NRO did not collect data on the total number of tests taken by day in dialysis patients and so we cannot assess the proportion of positive tests but rather the proportion of the hemodialysis population at risk. In addition we do not have an account of asymptomatic COVID-19 infections. However, strategies to reduce viral transmission did not differ between the two study periods of analysis apart from the introduction of the surgical mask protocol. At the time of writing, 3 months have passed following wave 1 of the COVID-19 pandemic without a case of COVID-19 in a haemodialysis patient in the Republic of Ireland.

Strategies such as universal surgical facemask use in hemodialysis units in addition to general population measures appear to restrict COVID-19 transmission and may be of use in areas of the world continuing suffering from a high burden of COVID-19 transmission.
