# Supplementary material for: The Impact of a National Surgical Mask Wearing Policy on COVID-19 Transmission in Haemodialysis Units in the Republic of Ireland
Source: Kidney Int Rep. 2021 Jan 1;6(3):810–2. doi: 10.1016/j.ekir.2020.12.031 (PMC7775609; doi:10.1016/j.ekir.2020.12.031)
Supplement: Supplementary File (PDF) [file mmc1.pdf]

Methods

The NRO collected incidence data pertaining to COVID-19 infection in End Stage Kidney Disease (ESKD) since the onset of the pandemic. We analysed national trends in COVID-19 positive cases over the course of the first wave of the pandemic in Ireland retrospectively.

Testing for SARS-CoV2 was performed by polymerase chain reaction on samples both nasal and Oropharyngreal swabs. All positive cases were recorded centrally by the NRO from March 15th 2020 onward, however negative tests were not recorded. Testing in Hemodialysis patients was precipitated not only by presence of symptoms consistent with SARS-CoV2 infection but also in any dialysis patient that became unwell for any reason in order to out rule atypical presentations.

This study assessed the impact of two interventions, an initial nationwide intervention by the Irish government on March 12th followed by a national surgical mask wearing policy in the haemodialysis units on April 7th 2020. For each period, daily positive cases were fitted by nonlinear regression. The change in overall slope was compared between the two time periods to determine the presence or absence of a statistically significant difference in trend. We also performed forecasting procedures to assess the likely trend that would follow the period up to April 7th 2020 without the mask policy introduction based on daily case rate up to that point assuming no change in trajectory. We also assessed the analysis by moving the date calipers 5 and 7 days forward from April 7th 2020 for surgical masking and 12th March for the national general population measures to assess for a lag effect of the intervention. *SAS version 9.4* was used for the data analysis.
